# Antimutagenic Effect of Medicinal Plants *Achillea millefolium* and *Bauhinia forficata In Vivo*

**DOI:** 10.1155/2013/893050

**Published:** 2013-12-29

**Authors:** Elisângela Düsman, Igor Vivian de Almeida, Ana Carolina Coelho, Thiago José Balbi, Lilian Tatiani Düsman Tonin, Veronica Elisa Pimenta Vicentini

**Affiliations:** ^1^Department of Biotechnology, Genetics and Cell Biology, State University of Maringá, Avenida Colombo 5790, Bloco H67 (11), Jardim Universitário, 87020-900 Maringá, PR, Brazil; ^2^Federal Technological University of Paraná, Rua Marcílio Dias 635, 86812-460 Apucarana, PR, Brazil

## Abstract

The investigation of traditionally used medicinal plants is valuable both as a source of potential chemotherapeutic drugs and as a measure of safety for the continued use of these medicinal plants. *Achillea millefolium* L. (AM) is an ancient remedial herb native to Europe that is used to treat wounds, gastrointestinal and hepatobiliary disorders, inflammation, headaches, and pain. *Bauhinia forficata* Link (BF), an Asiatic plant, is one of the most commonly used plants in folk medicine against diabetes. The aim of this study was to evaluate the cytotoxic and antimutagenic potential of aqueous extracts of AM and BF on bone marrow cells of Wistar rats treated *in vivo*. These plant extracts possess considerable antioxidant activity due to the presence of flavonoids and phenolic compounds. These compounds were determinants to noncytotoxic and antimutagenic/protective action of these plants, that reduced statistically the percentage of chromosomal alterations induced by the chemotherapeutic agent cyclophosphamide in simultaneous (AM, 68%; BF, 91%), pre- (AM, 68%; BF, 71%), and post-treatment (AM, 67%; BF, 95%). Therefore, the results of this study indicate that extracts of *A. millefolium* and *B. forficata* have antimutagenic potential and that their consumption can benefit the health of those using them as an alternative therapy.

## 1. Introduction

In recent decades, the amount of information about the use of tropical plants has increased considerably [1–3]. Brazil possesses great biodiversity and has a wealth of knowledge accumulated by local people who have direct access to nature and these resources [4]. Thus, folk medicine in Brazil, which is derived from a mixture of Brazilian indigenous cultures as well as European and African influences from the colonization period, is the basis for the knowledge regarding these traditionally used medicinal plants [3].

*Achillea millefolium *L. (Asteraceae), commonly known as yarrow, is an ancient remedial herb native to Europe and is used to treat wounds, gastrointestinal and hepatobiliary disorders, hemorrhages, spasmodic diseases, inflammation, headaches, and pain [5, 6]. Phytochemical screenings revealed that chemical constituents of *A. millefolium* (AM) present several secondary metabolites, including essential oils, sesquiterpenes, the alkaloid achilleine, steroids, triterpenes, and flavonoids, as presented by De Souza et al. [7]. A number of these substances had beneficial effects evidenced in several pathological conditions. Several traditional uses have been investigated and confirmed in experimental studies, including anti-inflammatory, analgesic, antiulcer, anxiolytic, hepatoprotective, hypotensive, and antiproliferative against human tumoral cells [7–13]. Despite therapeutic applications of the species, certain toxic effects in humans, including rhinitis, asthma, and contact dermatitis, have also been reported [10].

*Bauhinia forficata* Link (Leguminosae), an Asiatic plant well adapted to the Brazilian climate, is one of the most commonly used plants against diabetes in folk medicine. It is commonly known as “cow's paw” because of the shape of their leaves [14]. Phytochemical research has revealed that this plant contains alkaloids, tannins, mucilage, essential oil, cyanogenetic heteroglycosides, anthocyanins, saponins, catechols, and fixed volatile acids [15]. The hypoglycemic activity of *B. forficata* (BF) is given by the presence of glycosyl flavonoids, mainly kaempferitrin, present in the leaves of the plant and are readily extracted as from aqueous extracts [16]. Peroza et al. [17] studies have indicated that the aqueous extract of the leaves of BF is a potential source of natural antioxidants and may be helpful in the prevention of diabetic complications associated with oxidative stress. Besides these activities, BF also has action, antimicrobial, antiproliferative, and apoptotic [18, 19].

Because of the consumption of teas or aqueous extracts of AM or BF for the treatment of several conditions, including pain and diabetes, or as a coadjuvant with conventional drugs, it is of interest to determine whether the use of these plants has any benefit at the cytological and chromosomal levels, as no harm was found in our previous works. Studies involving the antimutagenic potential of medicinal plants are important because these plants can be used as a means of treatment without interfering in a normal lifestyle or result in the intake of an excessive number of synthetic substances. Thus, this study stands out from previously conducted studies because of its investigation of the evaluation of the potential antimutagenic/protector function of AM and BF aqueous extracts, against cyclophosphamide, a mutagenic chemotherapeutic agent, using as a test system Wistar rat bone marrow cells from *Rattus norvegicus*, *in vivo*.

## 2. Methodology

### 2.1. *In Vivo* Tests

#### 2.1.1. Treatment Solutions

Plants, *Achillea millefolium *L. (AM) (Exsiccate HUEM 12173, 07/06/2006, *lat*: −23.4253 *long*: −51.9386) and *Bauhinia forficata* Link (BF) (Exsiccate HUEM 12489 23/08/2006,* lat*: −23.4253 *long*: −51.9386), were collected on the day of treatment in Didactic Garden of the State University of Maringá (Maringá, PR, Brazil). The aqueous extract of each plant was prepared by infusion of fresh leaves (AM 35 g/L of water and BF 4.65 g/L of water). Boiling water was poured over AM or BF leaves, and after fifteen minutes, the infusion was leached and was allowed to cool. These concentrations correspond to ten times higher than the concentration normally used by the general population and showed no cytotoxic or mutagenic potential by Camparoto et al. [20] and Teixeira et al. [21].

#### 2.1.2. Wistar Rat Bone Marrow Cells

Six Wistar rats (*Rattus norvegicus*), three males, and three females for each group were obtained from the Central Vivarium of the State University of Maringá. Experiments were carried out using 35-day old rats weighing approximately 100 g (bw).

For antimutagenic analysis, plants were administered by gavage (1 mL/100 g bw) prior (2 h pretreatment), simultaneous to (simultaneous treatment), or after (2 h post-treatment) the application of an intraperitoneal injection of cyclophosphamide 1.5 mg/mL.

#### 2.1.3. Ethics Statement

During the experimentation period, the animals remained under controlled temperatures of ±25°C, with humidity at ±50% and with a photoperiod of 12 hours light/dark. Furthermore, all ethical principles, protocols, and regulations on experimentation with laboratory animals were used according to the standards established internationally and by the approved project by the Institutional Ethics Committee of State University of Maringá (UEM) and the Ethics Committee on Animal Use in Experimentation (CEAE)/UEM, following the Ethical Principles for Animal Experimentation established by the Brazilian College of Animal Experimentation (COBEA), as well as the specific treatment and collections protocols made to chromosomal aberration test.

#### 2.1.4. Chromosomal Aberration Test

A chromosomal aberration test was performed on the bone marrow cells from Wistar rats using Ford and Hamerton method [22], with some modifications. Mitotic cells were interrupted in metaphase with the intraperitoneal administration of 0.5 mL/100 g bw of colchicine (0.16%), half an hour before euthanasia. Analysis of the slides was performed by a light microscope, analyzing 100 cells in metaphase per animal, totaling 600 cells each for control and treatment groups.

Cells were assessed for the appearance of alterations, such as gaps, breaks, fragments, and others. Mitotic index (MI) for the cytotoxicity evaluation was calculated from 5,000 cells from each sex, totaling 10,000 cells per group. The MI was calculated as a percentage as follows: the number of dividing cells divided by the total number of cells present in the fields. Statistical calculation was performed using chi-square test (*n* = 6, *α* = 0.05).

### 2.2. Physicochemical Characterization

#### 2.2.1. Collecting and Drying Plants

The aerial parts (leaves and stems) of *A. millefolium* and *B. forficata* were collected in Didactic Garden of the State University of Maringá and dried at room temperature.

#### 2.2.2. Preparation of the Aqueous Extract

The infusion of each plant was prepared using 1.0 g of dried leaves in 100 mL of distilled water at approximately 100°C for 10 minutes. After that, the infusions were filtered through 100 mL volumetric flask and the meniscus calibrated with distilled water. The samples were stored under refrigeration for up to 24 hours before analyzes.

#### 2.2.3. Determination of Total Content of Phenolic Compounds

The determination of total phenolic content of the aqueous extract (10,000 *μ*g/mL) was performed according to the colorimetric method of Folin-Ciocalteu. An aliquot of 250 *μ*L of sample diluted in distilled water (1 : 10 v/v) was transferred to cuvettes containing 1,250 *μ*L of Folin-Ciocalteu reagent diluted in distilled water (1 : 10 v/v). The mixture was shaken and, after 5 minutes, 1 mL of a solution of sodium carbonate 4% (w/v) was added. After 2 hours resting, in absence of light, the absorbance of the samples was read at 740 UV-VIS spectrophotometer (Lambda 750, Perkin Elmer). The total phenol content was determined by interpolating with calibration curve constructed with standard gallic acid (concentrations between 5 and 80 mg/L) and expressed as mg gallic acid equivalents per gram of extract (mg GAE/g). All determinations were performed in triplicate.

#### 2.2.4. Total Flavonoids

The determination of flavonoids was performed according to the methodology described by Funari and Ferro [23] with adaptations. Concentration solutions were prepared at 10,000 *μ*g/mL of aqueous extracts. One mL of this solution was transferred to 10 mL volumetric flasks, in which 1.0 mL of 2% aluminum chloride-ethanol reagent was added, completing the volume with ethanol. After 30 minutes, readings were performed at 425 nm in a spectrophotometer (UV-VIS Lambda 750, Perkin Elmer). Before reading each extract, the unit was reset to zero with a solution containing all reagents except the aluminum chloride solution.

The construction of the calibration curve was prepared with a methanolic solution of rutin (0.5 mg/mL). Were removed, six aliquots to a 10 mL volumetric flasks containing 1 mL of 2% aluminum chloride in ethanol, completing the volume with ethanol. After 30 minutes, readings were at 425 nm. The results were expressed as mg rutin per gram of extract, or per gram of plant dry and percentage.

#### 2.2.5. Determination of Antioxidant Activity by the Method of Scavenging Free Radicals DPPH

Determination of antioxidant activity was performed by photocolorimeter of the DPPH (2,2-diphenyl-1-picryl-hydrazyl). One mL of infusion in different concentrations was added to 2.0 mL of a DPPH solution (40 *μ*g/mL, 0.10 mM in methanol). After remaining in the dark for 30 minutes, the absorbance was read at 515 nm on a UV-VIS spectrophotometer (Lambda 750, Perkin Elmer). The apparatus was reset with methanol. For each sample, we prepared a white replacing the DPPH solution by methanol 2.0 mL. The negative control was prepared by adding 1.0 mL of methanol to 2.0 mL of the DPPH solution. The inhibition percentage of DPPH radical was calculated according to the formula for Kulisic et al. [24] % inhibition = [(*A*_*c*_ − *A*_*a*_)/*A*_*c*_] × 100, where *A*_*c*_ = absorbance of the negative control and *A*_*a*_ = absorbance of the sample − absorbance of blank.

The amount of sample required to decrease the absorbance of DPPH in 50% was calculated graphically according to the method described by Kulisic et al. [24]. A graph of the percent of inhibition (% *I*) versus sample concentration was plotted, and the inhibition of 50% of DPPH was obtained by the equation of the straight line. BHT was used as reference compound and all determinations were performed in triplicate.

#### 2.2.6. Statistical Analysis

The results presented correspond to the chemical average of three replicates ± standard deviation. Were considered statistically different results of antioxidant activity showed that probability of the null hypothesis less than 5% (P < 0.05), applying ANOVA, followed by multiple comparisons by the Tukey test. Statistical analyzes were performed using STATISTICA version 8.0 (StatSoft, Inc., Tulsa, USA).

## 3. Results and Discussion

**Figure 1:**
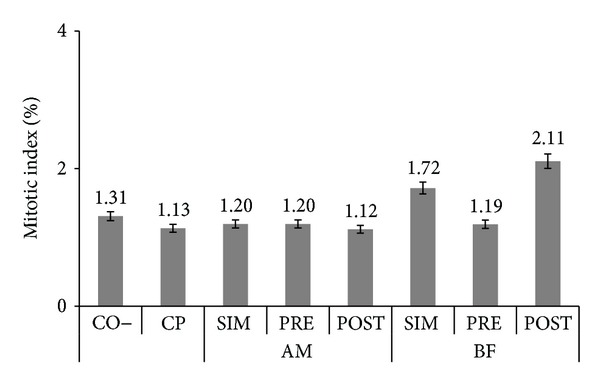
The mean percentage and standard deviation of the mitotic index for the negative (CO–) control group treated with *Achillea millefolium* L. (AM 35 g/L) or *Bauhinia forficata* (BF 4.65 g/L) simultaneous to (SIM), prior (PRE) and after (POST) the application of cyclophosphamide (CP 1.5 mg/mL).

The results of this study indicate that aqueous extracts of AM (35 g/L) and BF (4.65 g/L) administered by gavage (1 mL of solution/100 g bw) were not cytotoxic, that is, did not alter the mitotic index of the bone marrow cells of Wistar rats compared to the negative control (AM − SIM *χ* ^2^ = 0.09, PRÉ *χ* ^2^ = 0.05, and PÓS *χ* ^2^ = 0.08; BF − SIM *χ* ^2^ = 0.06, PRÉ *χ* ^2^ = 0.04, and PÓS *χ* ^2^ = 0.45) (Figure 1).

The data previously published by our laboratory [20, 21], also showing no cytotoxicity to Wistar rat bone marrow cells at different concentrations (AM − 3.5 g/L = 0.90% and 35 g/L = 1.40%; BF − 0.465 g/L = 1.57% and 4.65 g/L = 1.53%) corroborate these results.

Cavalcanti et al. [10] administered acute doses of up to 10 g/kg aqueous AM extract orally to rats and up to 3 g/kg intraperitoneally, with no deaths reported. In long-term studies, no signs of relevant toxicity in Wistar rats treated with aqueous AM extract at doses up to 1.2 g/kg/day by gavage for up to 90 days were reported; however, slight changes in liver weight and blood cholesterol and glucose levels were observed, though neither were correlated with the dose or period of exposure or were suggestive of toxicity. Pepato et al. [15] evaluated an orally administered decoction of BF in rats for its effects on the toxicity markers amylase (pancreas toxicity), creatine kinase (muscle toxicity), lactate dehydrogenase (muscle and liver toxicity), bilirubin (liver and biliary toxicity), and angiotensin-converting enzyme (renal microcirculation and kidney toxicity) and concluded that the decoction could be used in the treatment of diabetes, as it improves the diabetic condition without causing tissue toxicity, as shown by the biomarkers used.

Lin et al. [25] determined the antiproliferative activity of the extract of AM (2,000 *μ*g/mL) on different human liver tumors. The average inhibition of proliferation was 55.3% for the HepG2/C3A, SK-HEP-1, and HA22T/VGH lines and 20.3% for the lines Hep3B and PLC/PRF/5. Similarly, Huo et al. [26] isolated various compounds of AM, mainly flavonoids with antiproliferative potential on breast tumor and prostate cells (MCF7WT and PC-3, resp.). The plants used in the present study also show high levels of flavonoids and total phenols, as shown in Table 1.

Considering the results obtained, it appears that the observed antioxidant activity is directly correlated with the total content of phenolic compounds and flavonoids in the plants. This result is consistent with the report by Dröge [27], which stated that the phenolic compounds present in most plants inhibit the formation of free radicals.

Thus, due to the proven antioxidant activity of AM and BF and their phenolic components, it is important to assess whether the consumption of aqueous extracts of these medicinal plants can assist in the prevention or repair of cellular changes caused by the exposure to potentially mutagenic agents, in addition to the proven beneficial effects for health and well-being.

**Figure 2:**
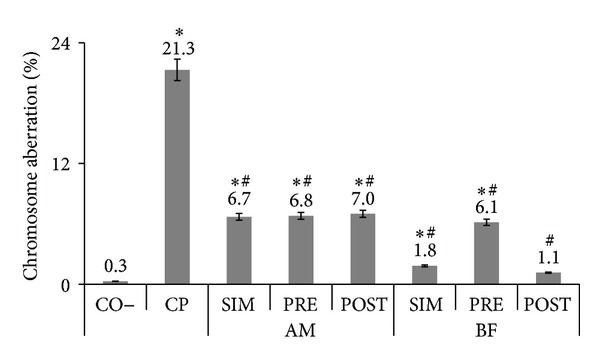
The mean percentage and standard deviation of the chromosome aberration for the negative (CO–) control group treated with *Achillea millefolium* (AM 35 g/L) or *Bauhinia forficata* (BF 4.65 g/L) simultaneous to (SIM), prior (PRE), and after (POST) the application of cyclophosphamide (CP 1.5 mg/mL). *Results statistically significant in relation to CO–. ^#^Results statistically significant in relation to CP.

The protective effect of aqueous extracts of these plants was determined by testing their antimutagenicity potential. Our results are supported by the fact that these plants showed no mutagenic activity in previous studies [20, 21], with no chromosomal alteration observed in the bone marrow cells of Wistar rats under the same conditions. In our case, the different treatments with AM significantly reduced (SIM *χ* ^2^ = 32.03; PRE *χ* ^2^ = 30.92; POST *χ* ^2^ = 29.21) the chromosomal alterations induced by the clastogenic agent cyclophosphamide by approximately 68.5% for the simultaneous treatment, 68.1% for the pretreatment, and 67.1% for the post-treatment (Figure 2). However, this reduction was statistically different from the negative control (SIM *χ* ^2^ = 136.53; PRE *χ* ^2^ = 140.83; POST *χ* ^2^ = 149.63).

It is noteworthy that *Achillea millefolium* and *Bauhinia forficata* plants presented similar types of chromosomal alterations, gaps and breaks, mainly chromatid, were predominant in simultaneous treatments (AM = 97.5%, BF = 81.8%), pre- (AM = 95.1%, BF = 35.1%), and post-treatments (AM = 92.8%, BF = 100%). These data show that some chemical agents, as the aqueous extracts of AM and BF, do not cause many double-stranded DNA breaks but rather other lesions, such as chromatid-type aberrations.

The three treatments with the BF aqueous extract showed a more effective antimutagenic action than the AM treatments. The simultaneous, pre-, and post-treatments significantly reduced (SIM *χ* ^2^ = 17.80; PRE *χ* ^2^ = 10.76; POST *χ* ^2^ = 19.04) the damage induced by cyclophosphamide by 91.4% for the simultaneous treatment, 71.1% for the pretreatment, and 94.6% for the post-treatment. In addition, post-treatment with the BF extract was able to reduce the damage induced by cyclophosphamide to the same level observed in the negative control, indicating an effective antimutagenic activity (SIM *χ* ^2^ = 7.80; PRE *χ* ^2^ = 114.46; POST *χ* ^2^ = 2.46) (Figure 2). The post-treatment mechanism of action occurs by an antimutagenic substance acting on the process that induces the formation of the mutation or the repair of DNA damage [28–30].

Ribeiro et al. [31] also proved the effective antimutagenic activity of caw's paw (*Bauhinia holophylla* Bong.) in hepatocellular carcinoma (HepG2). In this case, there was a decrease (76%) in the frequency of micronuclei induced by benzo[a]pyrene with caw's paw simultaneous treatment (7.5 *μ*g/mL).

According to Stavric [32], antimutagenic compounds can act at cellular level by enhancing the activities of enzymes involved in detoxification of mutagens, inhibiting the activities of enzymes involved in formation of mutagens metabolites, trapping of electrophils, scavenging reactive oxygen species, inhibiting metabolic activation, or protecting nucleophilic sites of DNA.

In this sense, it is possible that antimutagenic activity of AM and BF may be due to the combined action of their components, which has been particularly proven for the presence of flavonoids and phenolic compounds. Polyphenols, particularly flavonoids, have an ideal structure for scavenging free radicals and can also interact with the active groups of mutagens or protect the sites of DNA that would be affected by the mutagen [33–35]. These compounds and consequently the antioxidant activity of the extracts endow these plants with the ability to intercept the free radicals generated by cellular metabolism or exogenous sources, such as those resulting from the action of cyclophosphamide in this study, thereby preventing their damage to lipids, amino acids, proteins, polyunsaturated fatty acid double bonds, and DNA bases [36–38].

The aqueous extracts of PM and BF can act directly on compounds that induce mutations in DNA, chemically or enzymatically inactivating them, may inhibit the metabolic activation of promutagenic agents, or may scavenge reactive molecules, as explained by Kada et al. [28] and Kuroda et al. [30]. Thus, the considerable content of flavonoids in these two plants certainly contributed to their effective antimutagenic activity.

## 4. Conclusion

We observed that the aqueous extracts of plants (*Achillea millefolium* and *Bauhinia forficata*), which are routinely used for the treatment of pain and diabetes, have considerable antioxidant activity, show no cytotoxic activity, and may contribute to reducing the chromosomal damage induced by such chemotherapeutic agents as cyclophosphamide. Thus, the consumption of these plants can bring added benefits and protection to individuals undergoing treatment with cyclophosphamide or who use them for therapeutic purposes, improving their quality of life and health.

## Figures and Tables

**Table 1:** Content of total phenols (FT), flavonoids (FLV), and antioxidant activity (*I*), by inhibition of 50% of DPPH of *Achillea millefolium* and *Bauhinia forficata*.

Extract	FT (mg GAE/g)	FLV (mg rutin/g)	FLV (%)	*I* (*µ*g/mL)
AM	40.91 ± 0.20	13.90 ± 0.33	1.39	129.38 ± 5.46
BF	5.78 ± 0.17	4.91 ± 0.40	0.49	2,035.64 ± 30.04

Values represent the mean ± standard deviation (*n* = 3).
